# Getting under the skin of the menopausal hot flush: a protocol to examine skin function and structure in symptomatic postmenopausal women

**DOI:** 10.3389/fgwh.2025.1514960

**Published:** 2025-08-04

**Authors:** Kirsty A. Roberts, Abigail Doyle, Helen Jones, David A. Low

**Affiliations:** ^1^ Research Institute for Sport and Exercise Science, Liverpool John Moores University, Liverpool, United Kingdom; ^2^ Liverpool Centre for Cardiovascular Science at University of Liverpool, Liverpool John Moores University and Liverpool Heart & Chest Hospital, Liverpool, United Kingdom

**Keywords:** skin, menopause, hot flush, blood flow, sweating

## Abstract

**Introduction:**

The major pathophysiological symptom of the menopause affecting daily life is hot flushes, which are also associated with elevated cardiovascular disease risk. A hot flush is a sudden and intense heat sensation causing skin flushing and profuse sweating. Menopause-induced oestrogen deficiency could increase the sensitivity of skin blood vessels and sweat glands in postmenopausal women, which could result in more frequent and larger increases in skin blood flow in postmenopausal women consistent with hot flushes. Furthermore, oestrogen withdrawal could also alter the structure of the skin blood vessels and/or sweat glands which may also contribute to hot flushes. This trial aims to examine the function and structure of skin blood vessels and sweat glands in premenopausal and postmenopausal women.

**Methods and analysis:**

This is a single-centre multi-cohort observational study. Participants will attend the laboratory at Liverpool John Moores University (LJMU) on two separate occasions, ∼7 days apart. Visit 1 will consist of anthropometry, a blood sample and assessment of post-ganglionic skin blood vessel and sweat gland responsiveness via cutaneous microdialysis. At visit 2, participants will return for a skin punch biopsy. A between groups statistical analysis of the pre- and postmenopausal cohorts will be conducted in a blinded manner.

**Ethics and dissemination:**

The trial was approved by the North West - Greater Manchester South Research Ethics Committee (22/NW/0300) in the UK. The study adheres to The Declaration of Helsinki and is being conducted in accordance with the UK Policy Framework for Health and Social Care Research.

**Discussion:**

Identifying functional and/or structural changes in skin blood vessels or sweat glands in women with hot flushes would increase our understanding of their cause(s) and side effects, and help to design effective treatments, including interventions that can manipulate the activity of the skin blood vessels and/or sweat glands via pharmacological or non-pharmacological methods.

**Trial registration numbers:**

NCT06222073.

## Introduction

Menopause marks a crucial milestone in a woman's life and signals the end of the reproductive life cycle, usually occurring between the ages of 45 and 55 years. The cessation of oestrogen production has a direct effect on various organs in the body and women commonly experience wide-ranging menopausal symptoms, including sleep disturbance, bone loss, brain fog, weight gain, anxiety, depression and fatigue. Hot flushes (or vasomotor symptoms), the major pathophysiological symptom of the menopause, are experienced by ∼80% of women for up to several years (1) and can have a profoundly negative impact on quality of life (2, 3). A hot flush is an extreme thermoregulatory event, characterised by a sudden and intense feeling of heat, skin flushing/reddening and profuse sweating that can be triggered by environmental factors, such as heat stress, alcohol, caffeine and emotional stress, but often hot flushes occur spontaneously. The severity of hot flushes is associated with vascular dysfunction and increased cardiovascular disease risk through changes in cardiometabolic function (3, 4).

Hormone replacement therapy (HRT) can alleviate hot flushes, but is not suitable for all individuals, such as those with a medical history of hormone-receptive cancer, and HRT can also experience poor uptake due to historic links with breast cancer and an increased risk of cardiovascular disease (5). Fezolinetant, a non-hormonal neurokinin-3-receptor (NK_3_R) antagonist that dampens central thermoregulatory mechanisms associated with moderate to severe vasomotor symptoms, has recently been licensed for private prescription in the UK, but is not yet widely available. Few alternative treatments are available and consequently, many females suffer the debilitating symptoms of hot flushes due to a lack of therapies, and, ultimately, an incomplete understanding of the physiological mechanisms underpinning hot flushes.

The skin contains a vast array of neural, vascular and morphological structures and plays a crucial role in thermoregulation (6). The key events of a hot flush occur at the skin, namely, flushing/reddening and sweating. Elevations in skin blood flow and sweating include a series of inter-related steps (e.g., function) involving blood vessels and sweat glands (e.g., structure). Vasodilatory neurotransmitters or local substances bind to receptors on the skin blood vessels and sweat glands in order to cause vasodilation (increase in skin blood flow) and the release of sweat (7, 8). Previous work suggests that the increase in skin blood flow during a hot flush is initiated by elevations in neurotransmitters of vasodilator nerves (9) and nitric oxide (10). Other researchers have shown that calcitonin gene-related peptide (CGRP), a potent skin vasodilator (11, 12), is increased in the blood during hot flushes (13, 14). Oestrogen deficiency reduces levels of nitric oxide and CGRP (15, 16), possibly affecting skin blood vessel receptor sensitivity to these substances in postmenopausal women, potentially inducing more frequent and larger increases in skin blood flow consistent with hot flushes. Furthermore, women who experience hot flushes have enhanced skin blood flow responses to vasodilators (at the endothelium and smooth muscle) (17–19). Thus, skin blood vessels of symptomatic postmenopausal women might be overly sensitive to vasoactive substances that contribute to hot flushes, and the effect of oestrogen withdrawal on sweat gland receptors, and any potential alteration in their sensitivity, is also unknown.

The skin undergoes many morphological changes with advancing age (e.g., reduced collagen content and elasticity) which is accelerated with oestrogen withdrawal, resulting in reductions in collagen content, elasticity, water content and thickness (20). It is unknown if hypo-oestrogenism affects key vascular, sudomotor and/or neural structures in the skin, which also play a role in hot flushes. The number of sweat glands is generally constant across the lifespan in healthy individuals, but they are sensitive to repeated or a lack of stimuli. More specifically, the size of sweat glands and the number and density of the sympathetic nerve endings surrounding sweat glands can reduce as a result of decreased use (21). Similarly, the number of skin blood vessels can decrease with aging (22, 23), as they are also sensitive to repeated (or lack of) stimuli (24) leading to a reduction in both number and size when there are no recurring elevations in blood flow.

It is currently unknown whether the structure of skin blood vessels and/or sweat glands is affected by menopause or if they contribute to the occurrence of hot flushes. Furthermore, it remains uncertain whether there is a relationship between the function of skin blood vessels and/or sweat glands and the occurrence of hot flushes.

### Study aims

The overall aim of the study is to assess both function and structure of skin blood vessels and sweat glands in premenopausal and postmenopausal women. The specific objectives are:

1. To assess the responsiveness of skin blood vessels and sweat glands in premenopausal women and postmenopausal women with and without hot flushes.
2. To examine the structure of skin blood vessels and sweat glands in premenopausal women and postmenopausal women with and without hot flushes.

## Methods and analysis

### Study setting and recruitment plan

Recruitment (*n* = 36) will take place in the UK, commencing October 2024 for 12 months. The trial will end (last data collection from the last participant) in October 2025. A participant information sheet (PIS) will be given to potential participants, who will be recruited through local advertisement, social media websites and via lead members of local menopause support groups sharing the study information with their members.

### Sample size calculation

Based on previously reported differences in post-ganglionic skin blood flow responses between postmenopausal women with and without hot flushes (17–19), a power (1-β) of 0.9 and an effect size of 1, 24 postmenopausal women (12 who experience hot flushes and 12 who do not) and 12 premenopausal women will be recruited for this study.

### Eligibility criteria

Potential participants will exclude themselves based upon the detailed inclusion/exclusion criteria (Table 1) provided on recruitment material and the PIS. These criteria reflect the practical requirements of the study, to ensure that research findings are valid and to ensure the safety of participants. Participants will be consented and screened, consisting of medical history and details of current medications. Postmenopausal participants will complete a 7-day hot flush diary (25). Members of the research team will review completed screening information to confirm eligibility to participate.

**Table 1:** Detailed inclusion and exclusion criteria.

Detailed inclusion criteria		
Postmenopausal + Hot Flush	Postmenopausal	Premenopausal
• Female • Aged 45–65 years • Amenorrhoeic for >6 months • >4 Hot flushes per day • Not on medication or treatments to alleviate hot flushes e.g., HRT	• Female • Aged 45–65 years • Amenorrhoeic for >6 months	• Female • Aged 18–40 years • Eumenorrheic
AND		
• Healthy • Non-smoker/non-vaper • BMI 18–30 kg/m^2^ • No history of cardiovascular or respiratory disease • No history of metabolic diseases e.g., type II diabetes • Drink <14 units of alcohol per week • Participant is willing and able to give informed consent for participation in the study.		
Detailed exclusion criteria		
• Cannot readily read and understand English • Aged <18 years, 41–44 years or >65 years • Male • Current smoker/vaper • BMI <18 or >30 kg/m^2^ • Medical history of cardiovascular/respiratory disease • Medical history of metabolic disease e.g., type II diabetes • Drink >15 units of alcohol per week • Vaccination (<1 week) due to induced systemic inflammatory reaction • Local forearm infection • Allergy to local anaesthetic/Marcaine/amide-group anaesthetics • Currently pregnant, or planning on becoming pregnant • <6 Months postpartum or stopped breast feeding <1 month before recruitment • On medication or treatments to alleviate hot flushes or have taken such medication/treatments within the previous 6 months e.g., HRT		

### Outcome measures

#### Primary outcomes

The primary outcomes relate to the assessment of skin function and structure:

1. *Cutaneous microvascular function.* This will be assessed using the combination of cutaneous microdialysis and laser Doppler flowmetry.
2. *Cutaneous sudomotor function.* This will be examined using cutaneous microdialysis combined with capacitance hygrometry.
3. *Cutaneous blood vessel and sweat gland size.* This will be assessed using the skin punch biopsy technique and confocal microscopic imaging.

#### Secondary outcomes

Anthropometric data (e.g., height and weight), age and ethnicity will be collected. Resting blood pressure will be recorded at baseline and at intervals throughout the first visit. A single venous blood sample will be collected at the first visit to measure oestradiol and circulating pro-inflammatory markers.

### Experimental design

This is a parallel group design where participants will attend the laboratory on two separate occasions. The first visit will consist of anthropometric measurements and a blood sample, followed by assessment of post-ganglionic skin blood vessel and sweat gland responsiveness (cutaneous microdialysis). At the second visit (∼7 days later), participants will undergo a skin punch biopsy.

#### Visit 1: skin and sweat gland function

To minimise acute hormonal fluctuations, participants in the premenopausal group will attend for their first visit during the early follicular phase (days 1–5) of their menstrual cycle. To control for diurnal variation, testing will commence at 8.30am, lasting approximately 3–4 h. Participants will fast overnight and will be instructed to abstain from caffeine, alcohol, carbonated drinks for 12 h prior to testing, only drinking water. Participants will also be asked to avoid moderate/vigorous exercise for 24 h before testing. Ambient temperature in the laboratory will be controlled at 22°C–24°C (26).

##### Anthropometrics

Upon arrival, height and weight (Seca, Birmingham, U.K.) will be measured.

##### Blood pressure (BP)

Participants will rest in a seated position for ∼10 min prior to measuring their resting BP using an autosphygmomanometer (Dinamap V100, GE Healthcare, Chalfont St. Giles), with the BP cuff wrapped around the contralateral (dominant) arm to where skin function will be assessed. BP will then be measured in triplicate, leaving 1 min between successive measurements. BP will subsequently be recorded at 10 min intervals during the experimental protocol.

##### Blood sample

A single 8 ml blood draw will be taken from the antecubital fossa on the contralateral (dominant) arm to where skin function will be assessed. Blood samples will be centrifuged (2,500 RCF for 15 min at 5°C) and will be subsequently stored in aliquots at −80°C until analysis. Samples will be analysed using commercial enzyme-linked immunosorbent assays (ELISAs) for oestradiol (Merck, Dorset, U.K.), calcitonin gene-related peptide (CGRP; Bertin Bioreagent, Montigny le Bretonneux, France), prostaglandin 2E (Invitrogen) and a multiplex assay will be used to analyse circulating levels of inflammatory markers (MILLIPLEX, Merck, Dorset, U.K.).

##### Cutaneous microdialysis

**Figure 1:**
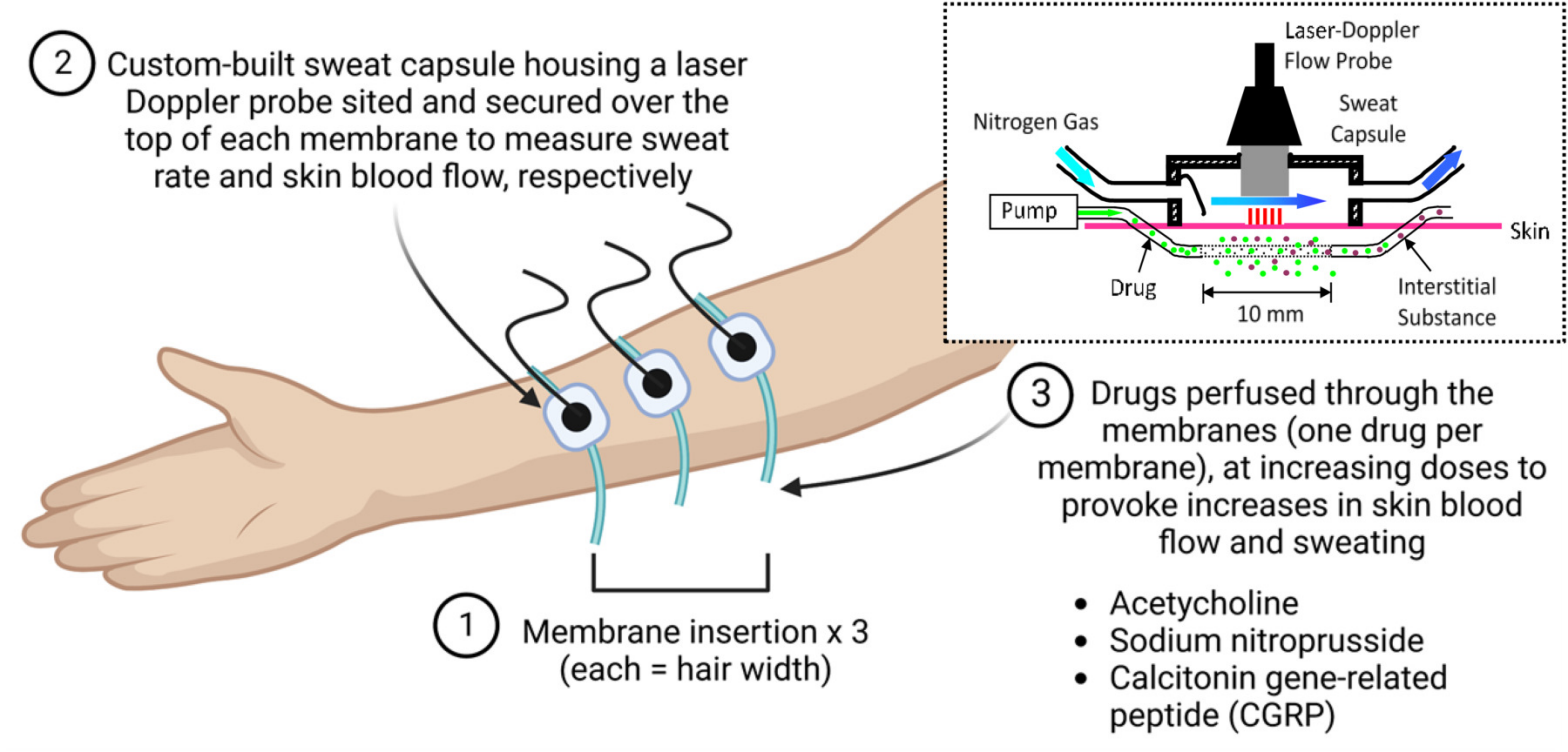
Illustration of the microdialysis technique and apparatus. Created in BioRender. Roberts, K. (2025). https://BioRender.com/ouwrev6.

Participants will rest in a supine position with ice applied topically to the non-dominant forearm for ∼10 min to acutely numb the non-dominant forearm, prior to insertion of a 25-gauge needle in the dermal space at a depth of 0.3–1.0 mm (27) (exiting 20 mm from the entry point) at three sites on the forearm, separated by >3 cm. An intradermal microdialysis membrane (Linear 30; CMA Microdialysis Ltd., Stockholm, Sweden) with 10 mm window, will then be threaded through the lumen of each needle, with each needle subsequently removed to leave the membrane *in situ* (Figure 1). Following successful placement of the membranes, lactated Ringer's solution will be perfused at 2 µl/min via a syringe infusion pump (Model 11 Plus, Harvard Apparatus, Natick, Massachusetts, USA). Following this, custom-made sweat capsules (3.0 × 2.0 × 1.2 cm) with integrated laser Doppler flowmetry probes (Perimed 413, Periflux 5001 System, Stockholm, Sweden) will be placed above each of the three embedded microdialysis membranes for the simultaneous quantification of skin blood flow (28) and sweat rate (SR) via capacitance hygrometry (HMT330, Vaisala, Vantaa, Finland) using compressed nitrogen gas at a flow of 300 ml/min (29). Absolute humidity of each capsule will be converted to SR from gas flow and the capsule surface area. Cutaneous vascular conductance (CVC) will be calculated [flux/mean arterial BP].

Following microdialysis membrane placement and allowing for its associated hyperaemic response to subside (≥90 min), baseline CVC and SR will be collected for 5 min. Each membrane will subsequently be randomly assigned and perfused with one of the following: increasing doses of CGRP (1 × 10^−11^–10^−4^; 8 doses at 10-fold increments) dissolved in Ringer's solution, acetylcholine (Ach; 1 × 10^−7^-1M; 8 doses at 10-fold increments) dissolved in Ringer's solution, or sodium nitroprusside (SNP; 1 × 10^−7^-50 mM; 8 doses at 10-fold increments) dissolved in Ringer's solution. Each dose will be administered for 7 min (1 min at a perfusion rate of 5 µl/min and 6 min at 2 µl/min). Following the last dose, 50 mM SNP will be perfused through each membrane for 10 min to initiate peak vasodilation at each of the assessed sites (29).

Data will be continuously sampled at 50 Hz (PowerLab, ADInstruments, Oxford, UK) and recorded online (LabChart, ADInstruments, Colorado Springs, Colorado, USA). For each dose, CVC and SR will be averaged at 10 s intervals, and the 60 s average around the maximum 10 s value from each stage will be selected for analysis (29).

A between groups statistical analysis of the pre- and postmenopausal cohorts will be conducted in a blind manner. Exploratory analyses using a linear mixed model with fixed effects of drug dose (cutaneous microdialysis) and/or group will be conducted. Dose-response curves for CVC and SR at each of the three microdialysis sites will be constructed using a nonlinear fitting technique, from which the effective concentration causing 50% of the maximal response (EC_50_) will be identified (Prism, GraphPad Software). *Post hoc* comparisons across drug doses or between groups will be completed using Bonferroni adjusted LSD. Effect estimates with a 95% confidence interval for differences between groups, as well as changes within groups over drug doses, will be reported.

#### Visit 2: skin structure and morphology

**Figure 2:**
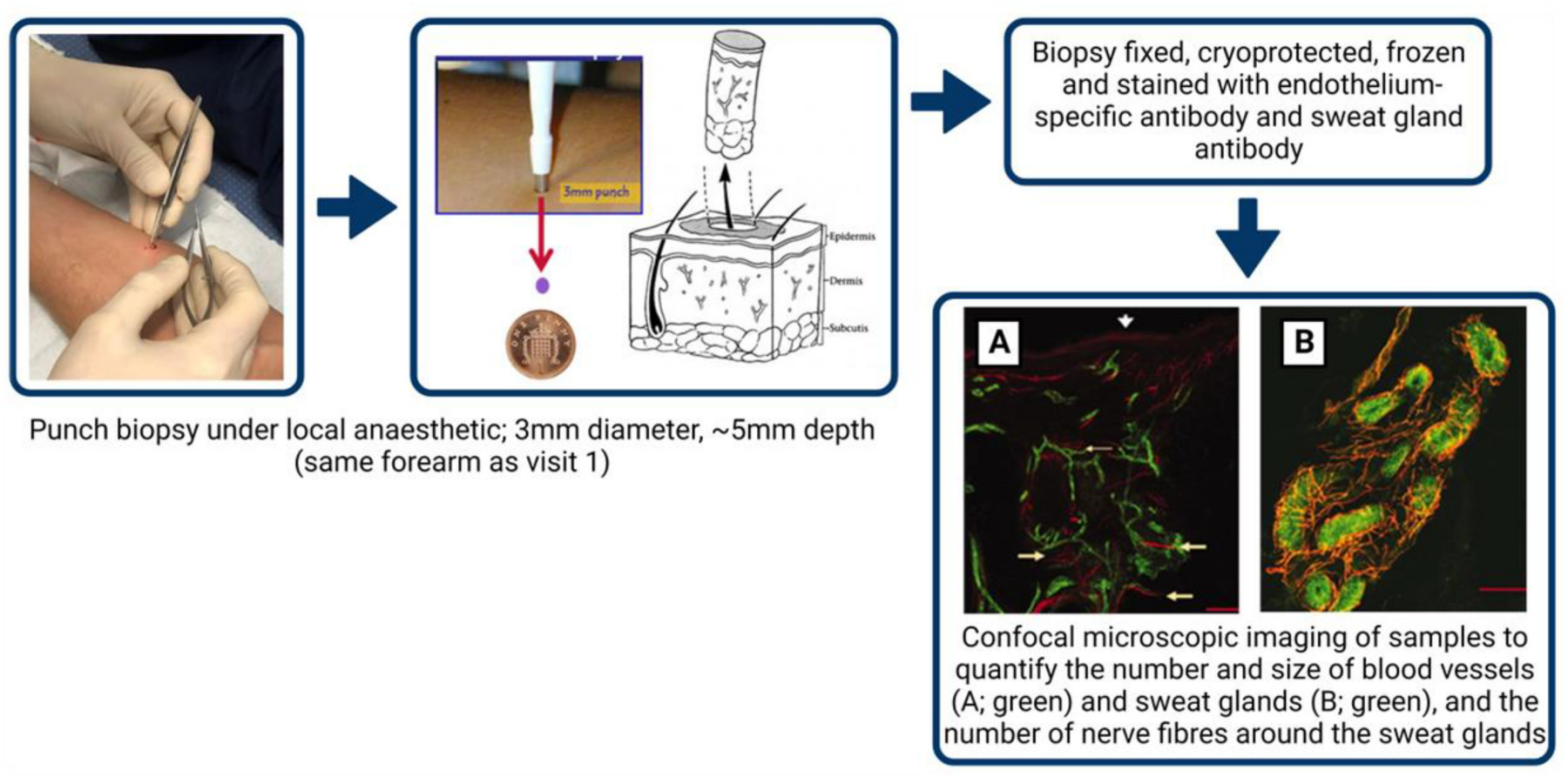
Skin punch biopsy methodology.

The minimally invasive skin punch biopsy technique, used for diagnostic investigation of skin blood vessels, sweat glands and nerves (30), will be performed according to international consensus guidelines (31, 32). Upon arrival, intradermal injection of local anaesthesia (Marcain) will be administered to a non-venous, non-hairy, tattoo-free location on the non-dominant forearm (site assessed in visit 1). A 3 mm diameter biopsy will be sampled using a punch biopsy tool (Stiefel, Maidenhead, U.K.) to an approximate depth of 5 mm (Figure 2). Biopsies will be placed immediately into a periodate-lysine-paraformaldehyde (PLP) fixative and stored for 24 h at 4°C, following which it will be cryoprotected in 15% sucrose for 24–72 h at 4°C, before being frozen in liquid nitrogen and stored at −80°C, adhering to all Human Tissue Act (2004) legislation and LJMU procedures.

Frozen samples will be cut (∼50 microns) and stained with fluorescein-labelled Ulex europaeus agglutinin I, an endothelium-specific antibody (33), and with anti-protein gene product 9.5 (1:1000, anti-PGP 9.5, Chemicon International Inc.), an antibody used to visualise nerve fibres around sweat glands and blood vessels in human skin.

Confocal microscopic imaging of samples (optical sections will be acquired at 2–4 μm intervals throughout the 50 μm section as a z-stack and will be projected in 3D images, Zeiss Axioplan 2, Germany) will subsequently be analysed using ImageJ software (32) to quantify the number and size of the stained blood vessels and the size of the secretory coils of the sweat glands, as well as the number of nerve fibres around the sweat gland within the samples (Figure 2). For between groups analyses (e.g., pre- vs. postmenopausal cohorts), data will be analysed using a one-way ANOVA.

### Study withdrawal

Each participant has the right to withdraw from the study at any time with no obligation to provide a reason. If provided, reasons for withdrawal will be retained, but personal data will be disposed of. Furthermore, participants may be withdrawn from the study by the research team at any time if the research team considers it necessary for any reason including:

• Ineligibility (either arising during the study or retrospectively having been overlooked at screening).
• Significant non-compliance with study requirements.
• Withdrawal of consent.

Withdrawal from the study will not result in exclusion of the participant's data from analysis, as all data will be pseudonymised. Withdrawn participants will not be replaced. Participants will be asked for their reasoning behind withdrawal, either via email or telephone call and the reason will be recorded in an end of study case report; participants are able to give no reason for withdrawal.

### Data management

Data will be collected and stored in accordance with the General Data Protection Regulation 2018. Direct access to data will be granted to the research team and host institution for monitoring and/or audit of the study to ensure compliance with regulations. Paper based data will be stored in a locked cabinet at LJMU, only accessible to the research team. All participants will be given a pseudonymised study code, which will be used for all stored data. An electronic document containing the link between a participants' name and study number will be stored in a password-protected file, only accessible to the research team, with a paper copy being stored in a locked cabinet at LJMU. The research team will be responsible for managing the administrative database (participant information) and trial data. Periodic checks will be conducted to verify the accuracy of the entered data against online records. Any errors will be documented and rectified. To ensure security, all data will be stored on computers that are password-protected and encrypted. Participant files will be stored for 5 years after the trial's completion, with access limited to the research team.

Our intended policy is that the research team will have exclusive access to the data for 12 months or until it is published. During this time, the data will be shared with any designated collaborators. Following this period, the data will be made publicly available through the LJMU Data Repository, under a permissive reuse license.

### Research governance and monitoring

A single research management group (RMG) will be responsible for overseeing the study and managing its day-to-day operations. The RMG will consist of medical professionals, senior and early career researchers who will also serve as the Data Monitoring Committee. The RMG will meet fortnightly to monitor the study's advancement, compliance with the protocol, safeguarding of participants and to evaluate any new data or relevant information from other sources. Furthermore, the RMG will maintain the study master file, respond to any questions about the study, ensure data security, quality and compliance, and conduct safety reporting.

### Patient and public involvement

The public and local groups will be involved in the study through their input in providing opinions on the protocol and all public-facing documentation (e.g., recruitment material) during the planning phase of the study.

## Discussion

The aim of this novel study is to examine both function and structure of skin blood vessels and sweat glands in pre- and postmenopausal women, who do and do not experience hot flushes. Findings from this work will reveal whether menopause is associated with altered function of skin blood vessels and sweat glands, such as an overt sensitivity to vasoactive substances that contributes to hot flushes. Furthermore, the study will examine whether menopause affects the structure of skin blood vessels and/or sweat glands, and whether a relationship exists between the function and/or structure of skin blood vessels and/or sweat glands, and the occurrence of hot flushes. Observation of any such changes in function and/or structure would increase our understanding of the causes and side effects of hot flushes, which would have important implications for patients and clinicians by helping in the design of effective treatments. Such therapies may include interventions that can manipulate the activity of the skin blood vessels and/or sweat glands via pharmacological and non-pharmacological methods.

Hot flushes, experienced by ∼80% of postmenopausal women, have a profoundly debilitating effect on daily activities and quality of life (1). Whilst menopausal hot flushes are widely recognised as being extreme thermoregulatory events with episodes typically lasting for several minutes and ranging from a few times per week up to several episodes per hour, their underpinning mechanisms are not well understood which has consequently limited scientific advances in hot flush therapies. Previous work has suggested mechanisms related to a narrowed null zone in the core temperature range of menopausal women reducing the temperature threshold for the onset of sweating, such that sweating will be activated and a hot flush triggered when an increase in core temperature surpasses the lowered threshold (34). However, this hypothesis is disputed as a further study observed that 49% of women did not experience a subtle increase in core temperature prior to a hot flush (35).

Central thermoregulatory and endogenous pyrogen mechanisms also represent plausible alternatives. NK_3_R stimulates the brain's thermoregulatory centre and preclinical research suggested heightened signalling of NK_3_R in menopausal hot flushes (36). Subsequent trials and treatment with a NK_3_R antagonist (e.g., Fezolinetant) demonstrated a marked reduction in moderate-to-severe vasomotor symptoms (37, 38), suggesting that one way of improving hot flushes is through modulating the NK_3_R pathway and hypothalamic-pituitary-gonadal (HPG) axis, a potential central driver of hot flushes. Endogenous pyrogens have also been implicated in hot flushes (39), although research is scant. Our work will contribute to the existing literature through analysing circulating inflammatory markers, including endogenous interleukin (IL)-1, IL-6, IL-8, IL-12 and tumour necrotic factor (TNF)-α.

Collectively, these studies and hypotheses represent potential central mechanisms responsible for hot flushes in postmenopausal women. Conversely, our findings hope to reveal important insight as to whether local changes in peripheral sites, such as the skin, contribute to the occurrence of hot flushes; for instance, postmenopausal women who experience hot flushes exhibiting greater responsiveness of skin blood vessels and sweat glands.

### Study limitations

Whilst this protocol has been designed to comprehensively evaluate the function and structure of skin blood vessels and sweat glands in postmenopausal women, there are some limitations to the study. Firstly, being an observational study, it does not allow causal conclusions to be drawn between menopause, hot flushes and any changes in skin function and/or structure. Rather, causal relationships will require investigation through longitudinal and/or intervention studies. Secondly, as the sample size is relatively small (*n* = 36), statistical power may be limited, particularly in detecting small differences between groups. Furthermore, recruitment from a single geographical area in the U.K. may mean that findings are not generalisable to the wider population or across ethnic groups. Participants will subjectively report hot flush incidence and severity using a 7-day diary (25) which may be susceptible to perception and/or reporting bias. However, the questionnaire is validated, demonstrating good reliability and consistency against controlled studies. The skin punch biopsy will provide important information about skin structure, yet the single forearm biopsy means that any morphological changes in this limited area, will not necessarily reflect systemic changes in skin structure. Finally, single-time sampling means that measurements will not allow for potential subtle changes according to diurnal rhythms, or hormonal fluctuations, which are beyond the scope of this study.

## Data Availability

Following publication of the study’s papers, pseudonymised data will be made available for sharing with other investigators. Data will be shared through the LJMU Data Repository (http://opendata.ljmu.ac.uk/), a secure institutional data repository, managed by LJMU Library Services. A DOI will be generated for datasets as they are deposited to the repository. Data will be stored in this repository for a minimum of 10 years, or for 10 years from the last date of access.
